# Morphological and Volumetric Analysis of Left Atrial Appendage and Left Atrium: Cardiac Computed Tomography-Based Reproducibility Assessment

**DOI:** 10.1371/journal.pone.0101580

**Published:** 2014-07-02

**Authors:** Mikko Taina, Miika Korhonen, Mika Haataja, Antti Muuronen, Otso Arponen, Marja Hedman, Pekka Jäkälä, Petri Sipola, Pirjo Mustonen, Ritva Vanninen

**Affiliations:** 1 Department of Clinical Radiology, Kuopio University Hospital, Kuopio, Finland; 2 Unit of Radiology, Institute of Clinical Medicine, University of Eastern Finland, Kuopio, Finland; 3 Heart Center, Kuopio University Hospital, Kuopio, Finland; 4 NeuroCenter, Kuopio University Hospital, Kuopio, Finland; 5 Unit of Neurology, Institute of Clinical Medicine, University of Eastern Finland, Kuopio, Finland; 6 Department of Cardiology, Keski-Suomi Central Hospital, Jyväskylä, Finland; Shanghai Institute of Hypertension, China

## Abstract

**Objectives:**

Left atrial appendage (LAA) dilatation and morphology may influence an individual's risk for intracardiac thrombi and ischemic stroke. LAA size and morphology can be evaluated using cardiac computed tomography (cCT). The present study evaluated the reproducibility of LAA volume and morphology assessments.

**Methods:**

A total of 149 patients (47 females; mean age 60.9±10.6 years) with suspected cardioembolic stroke/transient ischemic attack underwent cCT. Image quality was rated based on four categories. Ten patients were selected from each image quality category (N = 40) for volumetric reproducibility analysis by two individual readers. LAA and left atrium (LA) volume were measured in both two-chamber (2CV) and transversal view (TV) orientation. Intertechnique reproducibility was assessed between 2CV and TV (200 measurement pairs). LAA morphology (A = Cactus, B = ChickenWing, C = WindSock, D = CauliFlower), LAA opening height, number of LAA lobes, trabeculation, and orientation of the LAA tip was analysed in all study subjects by three individual readers (447 interobserver measurement pairs). The reproducibility of volume measurements was assessed by intra-class correlation (ICC) and the reproducibility of LAA morphology assessments by Cohen's kappa.

**Results:**

The intra-observer and interobserver reproducibility of LAA and LA volume measurements was excellent (ICCs>0.9). The LAA (ICC = 0.954) and LA (ICC = 0.945) volume measurements were comparable between 2CV and TV. Morphological classification (ĸ = 0.24) and assessments of LAA opening height (ĸ = 0.1), number of LAA lobes (ĸ = 0.16), trabeculation (ĸ = 0.15), and orientation of the LAA tip (ĸ = 0.37) was only slightly to fairly reproducible.

**Conclusions:**

LA and LAA volume measurements on cCT provide excellent reproducibility, whereas visual assessment of LAA morphological features is challenging and results in unsatisfactory agreement between readers.

## Introduction

Stroke is the leading cause of long-term disability and the second highest cause of mortality globally [1]. The currently recognized mechanisms for ischemic stroke or transient ischemic attack (TIA) are embolism, local vascular thrombosis, and decreased brain tissue perfusion due to other reasons [2]. Cardioemboli are derived mainly from the left atrial appendage (LAA) [3]. Despite atrial fibrillation (AF) being the most common risk factor for cardioembolism, it is not always recognized during acute cardioembolic events [4]. Over one-third of ischemic strokes/TIAs remain undetermined. The role of LAA enlargement in the etiology of these strokes may be underestimated, and cryptogenic stroke/TIA has been associated with enlarged LAA volumes [5], [6]. The role of the LAA in thrombus formation and stroke risk has been studied using multiple imaging modalities [7]–[13]. Recent magnetic resonance imaging (MRI) studies have shown that LAA volume is strongly associated with an increased prevalence of prior stroke/TIA, and the risk differs between LAA morphologies [12], [13]. Percutaneous LAA occlusion has the advantage of being a minimally invasive treatment for patients in whom long-term anticoagulation treatment is deemed unsuitable and may be equivalent to treatment with oral anticoagulant agents in those individuals considered at moderate-to-high risk of thromboembolism [14]. Increasing evidence indicates that the upper quintile of LAA volume is a powerful predictor of stroke risk [15]. Novel low-dose computed tomography (CT) scanners may increase pressure to use cardiac CT (cCT) for both etiological assessment and risk stratification [16]. Although 2D transesophageal echocardiography (TEE) is currently the most commonly used imaging modality for preoperative assessment, the use of 3D approaches have shown to provide additive information in cases of complex morphology of the LAA [17].

The value of a measurement method is largely dependent on its ability to provide reproducible and reliable results. To the best of our knowledge, the magnitude of these errors in the assessment of LAA volume and morphology by cCT has not been fully elucidated. We analysed the intra-observer and interobserver reproducibility of LAA and left atrium (LA) volume measurements in the transversal (TV) and two-chamber view (2CV) orientations and evaluated the repeatability of visual assessments of the morphological features of the LAA.

## Materials and Methods

The study was part of the EMBODETECT project [6]. The study has been approved by the Kuopio University Hospital Research Ethics Board and all clinical investigation have been conducted according to the principles expressed in the Declaration of Helsinki. Written informed consent was obtained from the participants or their legally authorized representative if a patient was unable to provide consent due to impaired mental or physical function caused by stroke.

### Study Design and Population

Acute stroke/TIA patients admitted to our university hospital between March 2005 and November 2009 were evaluated as candidates for this cCT study. The neurologists involved in the study recruited 162 patients (47 females; mean age 60.9±10.6 years) with suspected cardioembolic stroke/TIA without known atrial fibrillation. Thirteen patients were excluded for the following reasons: cCT image quality was not appropriate for analysis of LAA size (n = 4), ECG synchronization was not used for cardiac imaging (n = 3), contrast media injection failed (n = 2), use of contrast media was contraindicated due to decreased renal function (n = 1), or the patients refused to participate after giving informed consent (n = 3). The remaining 149 patients were included in the present study.

### CT Imaging

Contrast-enhanced cCT was performed with a 16-slice (n = 113 patients) or 64-slice (n = 36 patients) scanner (Somatom Sensation 16 and Somatom Definition AS; Siemens Medical Solutions, Forchheim, Germany). Prior to imaging, β-adrenoreceptor antagonists were not administered. The aortic arch and cervical and intracranial arteries were scanned first, immediately followed by the ascending aorta and heart. When using the 16-slice scanner, contrast agent was injected through an 18-gauge catheter into the antecubital vein at 5 mL/s, followed by a 50-mL injection at 2 mL/s and subsequent 20-mL saline chaser. With the 64-slice scanner, 100 mL of contrast agent (350 mgI/mL) was injected at 5 mL/s, followed by a 20-mL injection at 2 mL/s and subsequent 20-mL saline chaser. Cardiac imaging was performed during mid-diastole in all study subjects. In the 16-slice scanner, collimation was 16×0.75 mm, the rotation time 0.42 s, and tube potential 120 kV; the current was set to 500 mAs for the first 80 patients and reduced to 250 mAs thereafter. In the 64-slice scanner, collimation was 64×0.6 mm, the rotation time 0.33 s, and tube potential 120 kV; the reference current was set using commercially available tube current modulation software (CAREDose4D, Siemens Medical Solutions) at 160 mAs for the first 15 patients and then reduced to 100 mAs. Mid-diastolic 0.75 to 1.0-mm-thick slices with 20–25% overlap were reconstructed.

### LAA and LA Volume Measurements

Quantitative image analysis was performed on an IDS5 diagnostic workstation (version 10.2P4; Sectra Imtec, Linköping, Sweden) using magnified images on 1024×768 and 1600×1200 displays. The oblique sagittal 2CV was obtained perpendicular to the mitral valve and parallel to the cardiac septum, and set from the left ventricle (LV) apex to the centre of the mitral annulus. The TV was obtained from horizontal slices. Due to contrast agent injection, the LAA was seen as a hyperdense structure surrounded by hypodense pericardial fat with high tissue contrast between the anatomical structures. The entire LAA was fully opacified with contrast media in all study subjects. The LAA borders were traced manually on the 2CV and TV using the mitral valve annulus as a landmark to differentiate the LA from the LV. The 2CV stack and localizer tool were used to differentiate the LAA orifice from the LA in the TV. Planimetration of the entire LAA comprised a mean 10.4±2.0 slices in the 2CV and 10.8±1.9 slices in the TV, whereas planimetration of the entire LA comprised a mean 20.0±3.2 slices in the 2CV and 17.6±2.9 slices in the TV. Volumes were calculated using Simpson's method by multiplying the area of each manually traced LAA and LA by the section thickness (3 mm) and summing the volumes of the separate sections.

### Intra-observer and Interobserver Reproducibility of Volume Measurements

**Figure 1:**
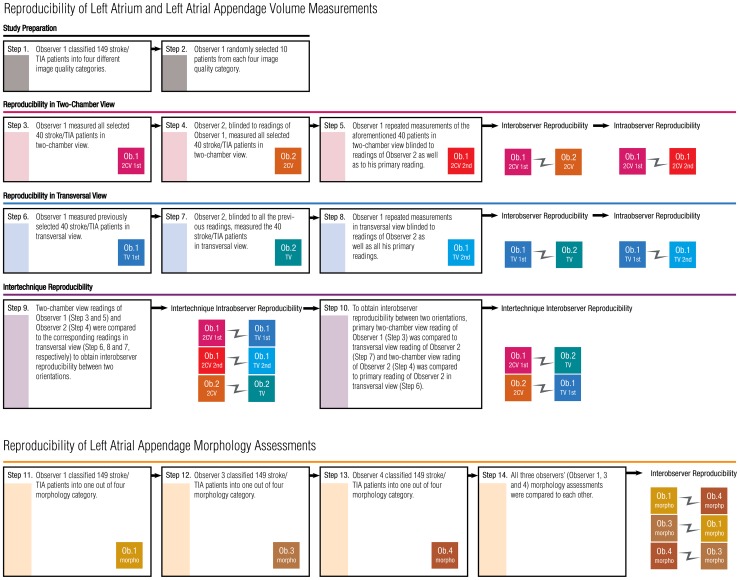
Study design for reproducibility analyses.

All observers were guided by a cardioradiologist. Observer 1 performed planimetration in both the 2CV and TV. Image quality was rated as excellent, good, moderate, or poor. Forty patients were selected for reproducibility analysis based on representative image quality, resulting in 10 scans being selected from each of the four categories. To calculate intra-observer repeatability, the observer reconstructed new slices in both orientations and repeated the LAA and LA measurements one month later while blinded to the previous measurements. For interobserver reproducibility, Observer 2 reconstructed new slices and analysed the LAA and LA volumes of the same 40 patients in both orientations (Figure 1).

### LAA Morphology Analysis

As originally described by Wang et al [13], [18], the shape of the LAA was classified as: A) Cactus, B) ChickenWing, C) WindSock, or D) CauliFlower [19]. The Cactus is defined as a dominant central lobe, limited overall length, and one or more secondary lobes; ChickenWing as a main lobe that bends from the proximal middle part of the LAA; WindSock as one dominant lobe with several secondary, or even tertiary, lobes; and CauliFlower as limited overall length and complex internal structures. The opening height of the LAA compared to the mitral annulus, the number of LAA lobes, the amount of intra-LAA trabeculation, and the orientation of the tip of the LAA were classified into three categories.

### Interobserver Reproducibility of LAA Morphological Assessments

Three independent observers (Observers 1, 3, and 4) reconstructed the 2CV and visually analysed LAA morphology while blinded to collateral readings. Consequently, the reproducibility of visual classifications of LAA morphology was assessed with 447 independent classifications made by three observers (Figure 1).

### Statistical Analysis

Continuous variables are presented as mean ± standard deviation (SD) and categorical variables as absolute values and percentages. Spearman's correlation coefficient was used to investigate the associations between continuous variables. Significance was set at *P*<0.05 and highly significant at *P*<0.01. In volumetric analyses, intraclass correlation coefficients (ICC) were calculated using a two-way mixed-effects model with absolute agreement to assess the interobserver and intra-observer reproducibility in both orientations and between different orientations across all four image quality categories. ICC values from 0.0 to 0.2 were considered negligible, from 0.2 to 0.4 very low, from 0.4 to 0.7 moderate, from 0.7 to 0.9 high, and from 0.9 to 1.0 very strong. Interobserver and intra-observer coefficients of variability (CV%) were assessed to evaluate the volume diversity across image quality categories for both readers in both orientations and between different orientations. CV% was calculated by dividing the SD of the differences by the mean of the parameter under consideration. In the current study, group variability with a CV% <8% was considered homogenous, from 8 to 10% average, and >12% diverse [20]. Based on a normal distribution in the Kolmogorov-Smirnov test, the Mann-Whitney U test was used to compare the ICC and CV% in dichotomous variables and the Kruskal-Wallis test in polytomous variables. The correlation between the ICC and CV% was measured to evaluate whether reproducibility is associated with volume diversity. Bland-Altman plots were created to exclude systematic bias depending on LAA and LA volume or the observers. Cohen's kappa was used to quantify the interobserver measurements for LAA morphology assessments; kappa values from 0.0 to 0.2 were considered slight, from 0.2 to 0.4 fair, from 0.4 to 0.6 moderate, from 0.6 to 0.8 substantial, and from 0.8 to 1.0 near perfect [21]. Data were analysed using SPSS for Windows (version 19, 1989–2010 SPSS Inc., Chicago, USA).

## Results

### Patient Characteristics and Image Quality

The characteristics of all 149 patients included in the morphological analyses and the 40 patients included in different types of reproducibility analyses are shown in Table 1. The mean radiation dose was 10.0±3.5 mSv (range 2.0–16 mSv). Image quality was rated as excellent in 22 (61%), good in 8 (22%), moderate in 6 (17%), and poor in 0 (0%) of the 36 patients imaged with the 64-slice scanner, and in 8 (7%), 34 (30%), 45 (40%), and 26 (23%) of the 113 patients imaged with the 16-slice scanner, respectively. All investigated patients were in sinus rhythm during cCT scan. There was no association between hearth rate and CV% nor a correlation between image quality and heart rate.

**Table 1:** Clinical Characteristics of Patients.

Characteristic	Morphology analysis n = 149	Volumetric analysis n = 40	*Sig.*
Age, yr	60.9±10.6	60.1±9.7	ns.
Females, n (%)	47 (31.5)	11 (38.2)	ns.
Body mass index, kg/m^2^	28.1±4.4	29.2±4.0	ns.
Body surface area, m^2^	2.0±0.2	2.0±0.2	ns.
Caucasian race, n (%)	149 (100.0)	40 (100.0)	ns.
Hypertension, n (%)	88 (59.1)	21 (52.5)	ns.
Hyperlipidaemia, n (%)	61 (40.9)	18 (45.0)	ns.
Diabetes, n (%)	22 (17.8)	3 (7.5)	ns.
Smokers, n (%)	38 (25.5)	7 (17.5)	ns.
Prior stroke, n (%)	29 (19.5)	5 (12.5)	ns.
Prior myocardial infarction, n (%)	19 (12.8)	4 (10.0)	ns.
Sinus rhythm during imaging, n (%)	149 (100.0)	40 (100.0)	ns.
Heart rate, beats per minute	67.3±11.2	66.7±10.0	ns.
PAF in 24 hour ECG Holter, n (%)	38 (25.5)	1 (2.5)	0.033
Thrombus in LAA, n (%)	3 (2.0)	0 (0.0)	ns.

Sig = significance; ns =  not significant in level P<0.05; PAF = paroxysmal atrial fibrillation.

### Reproducibility of LAA and LA Volume Measurements

The results of the interobserver and intra-observer reproducibility analyses of LAA and LA volume measurements are shown in Table 2. Based on ICC values, reproducibility was very high despite variations in image quality. Volumetric measurements in 2CV and TV resulted in excellent comparability (Table 3).

**Table 2:** Interobserver and Intra-observer Reproducibility Analysis of Left Atrial Appendage (LAA) and Left Atrium (LA) Volumes.

	Two-Chamber View								Transversal View							
	LAA interobserver		LAA intra-observer		LA interobserver		LA intra-observer		LAA interobserver		LAA intra-observer		LA interobserver		LA intra-observer	
	ICC	CV%	ICC	CV%	ICC	CV%	ICC	CV%	ICC	CV%	ICC	CV%	ICC	CV%	ICC	CV%
Excellent	0.929	10.1	0.978	6.0	0.977	4.3	0.998	1.7	0.976	5.3	0.939	8.1	0.967	8.3	0.995	3.3
Good	0.970	13.3	0.988	9.1	0.987	4.6	0.988	3.6	0.943	21.0	0.988	8.2	0.948	8.9	0.979	5.8
Average	0.927	18.2	0.984	5.5	0.946	11.1	0.996	3.1	0.972	8.6	0.984	8.0	0.970	9.0	0.998	2.8
Poor	0.978	12.3	0.995	5.0	0.972	6.9	0.989	5.2	0.946	17.2	0.954	13.7	0.905	14.5	0.985	5.5
Total	0.960	13.5	0.988	6.4	0.996	6.7	0.992	3.4	0.953	13.0	0.970	9.5	0.944	10.2	0.989	4.3

Forty measurements pairs included 10 patients from each image quality category.

**Table 3:** Intertechnique Reproducibility of Left Atrial Appendage and Left Atrium Volume Measurements.

	Measurement Pairs	Left Atrial Appendage		Left Atrium	
Image Quality	N	ICC	CV%	ICC	CV%
Excellent	50	0.917	9.8	0.965	7.3
Good	50	0.963	13.5	0.944	8.0
Average	50	0.957	12.1	0.949	9.8
Poor	50	0.953	14.1	0.930	10.6
Interobserver	80	0.949	13.0	0.931	9.4
Intra-observer	120	0.957	12.0	0.955	8.6
Total	200	0.954	12.4	0.945	8.9

Two hundred measurement pairs: 80 pairs with different observers and 120 pairs with the same observer.

Combining all reproducibility analyses, the CV% values did not indicate significant volume diversity in interobserver and intra-observer analyses, intertechnique analyses between 2CV and TV, or analyses according to image qualities (Table 2). A significant (correlation coefficient  = −0.817; *P*<0.01) negative correlation was observed between the ICC and CV%.

**Figure 2:**
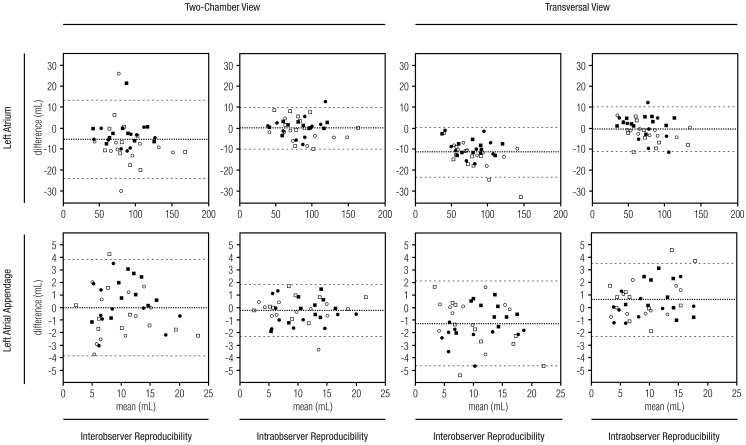
The reproducibility of volume measurements. Bland-Altman plots show the reproducibility of 40 left atrium and 40 left atrial appendage volume measurements for the same observer and different observers in two-chamber view and the transversal view. The X-axis shows the mean of two volume measurements and the y-axis shows a difference between respective measurements. The middle line corresponds to the mean difference and the outer lines correspond to the 95% confidence interval. Filled squares (▪) indicate cardiac computed tomography images with excellent image quality; filled circles (•) indicate good image quality; empty circles (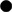), moderate image quality; and empty squares (□), poor image quality. Volume size and image quality had no significant influence on interobserver or intra-observer measurements in either orientation.

**Figure 3:**
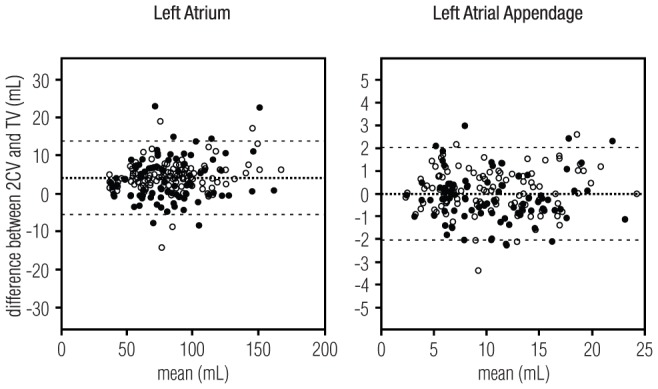
The intertechnique reproducibility of volume measurements. Bland-Altman plots show the intertechnique reproducibility of 200 left atrium and 200 left atrial appendage volume measurements performed in two-chamber view or transversal view. The X-axis shows the mean of two volume measurements and the y-axis shows the difference between respective measurements. The middle line corresponds to the mean difference and the outer lines correspond to the 95% confidence interval. Filled circles (•) indicate 80 interobserver measurement and empty circles (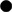) 120 intra-observer measurements performed in the two orientations. Volume size and observer had no significant influence on the measurements.

The Bland-Altman plots in Figure 2 show that the reproducibility of LAA and LA volume measurements does not depend on the volume. No systematic difference between image quality or measurement orientations was observed. The Bland-Altman plots in Figure 3 show that no systematic bias was introduced due to different readers for volume measurements in the 2CV or TV orientation.

### Reproducibility of LAA Morphological Assessments

Cohen's kappa value indicated fair agreement between observers in the assessment of LAA morphology (ĸ = 0.24) and LAA tip orientation (ĸ = 0.37), and slight agreement between observers in the assessment of LAA opening height (ĸ = 0.1), the number of LAA lobes (ĸ = 0.16), and the amount of trabeculation (ĸ = 0.15).

## Discussion

The main finding of the current study was that LAA and LA volume measurements using cCT are highly reproducible and comparable between observers, despite differing image quality, variation in LAA or LA volumes, and differing measurement orientations. Visual classification of LAA morphology into four classes (Cactus, ChickenWing, WindSock, or CauliFlower) and the evaluation of LAA tip orientation had fair interobserver reproducibility. However, the more detailed visual classifications of other morphological features were unsatisfactory, resulting in only slight agreement between the readers.

The detection of an enlarged LAA in patients with acute ischemic stroke/TIA may be useful in the risk stratification of recurrent stroke. Recent multicentre studies have shown that patients in the highest LAA volume quintile are at an increased risk of cardioembolic events [15]. In addition, increased LAA and LA volumes have been detected in patients with AF and paroxysmal AF, but also cryptogenic patients with no obvious etiology for stroke/TIA [6], [12], [22], [23]. Some studies have also speculated whether certain LAA morphology types increase the risk for thrombus formation [13], [18], [24].

In the future, not only stroke/TIA, but also AF diagnostics and treatment, may take advantage of an accurate evaluation of LAA or LA volume [22]–[25]. To be clinically feasible, these assessments should be highly reproducible. Though considered haemodynamically more vivid, the main interest thus far has been on LA volumetry. Studies on the reproducibility of LAA volume measurements are scarce, despite increasing interest in LAA volumes [26]. Previous studies have also implied a poor correlation between LAA and LA volume, underlining the importance of separate LA and LAA evaluations [27]–[29]. In a previous retrospective study of 74 patients undergoing 64-slice cCT, the end-diastolic volume measurements showed significant reproducibility when evaluated with the Pearson correlation [30]. The different statistical approach makes a comparison with our results difficult. On the other hand, the Bland Altman plots did not reveal a significant bias associated with LA size [30]. In another previous study, 3D threshold-based end-diastolic volume measurements yielded ICC values of 0.961–0.997 for intra-observer and 0.867–0.926 for interobserver analyses [31], which are highly compatible with our results. Our current study was carried out with relatively old scanners. More novel 320-slice CT scanners visualize the boundaries of the LA with more advanced spatial resolution, comparable to 1.5-tesla MRI [32], [33]. In a study comparing 320-slice cCT and transthoracic 2D and 3D echocardiography (TTE), the methods showed a strong positive correlation, but the absolute value of the LA volume on cCT was significantly larger than that determined by either 3D or 2D TTE, which can be due to an overestimation by cCT, underestimation by TTE, or both [34]. LAA morphology is widely used for the pre-evaluation of percutaneous LAA closure procedures [35]. The main exclusion criterion for this treatment is the presence of thrombus in the LAA [36]. TEE provides a more comprehensive tool to assess decreased blood flow velocity in LAA [14]. Nevertheless, detecting solid LAA thrombi seems feasible also with cCT [37]. According to our results, LAA volume measurements from different orientations in cCT are comparable. Although reproducible, manual LAA volume measurements are highly time-consuming, resulting in an urge to create automatized segmentation tools for clinical use. Our results indicate that an automatized segmentation tool may be based on transversal images provided automatically by the CT scan and that no specific stacks need to be formed for segmentation. LAA volumes in the 2CV seem to vary only slightly more between the different readers.

Our results yielded only slight to fair reproducibility for the visual classification of morphological features of the LAA. This result differs from the highly reproducible interobserver assessments of morphology reported by Di Biase et al [13]. They found no significant bias in classifying LAA morphology on CT (ĸ = 0.84). The classification method in Kimura et al [17] resulted in even higher interobserver agreement (ĸ = 0.901). Nevertheless, similar to our results, Khurram et al [24] reported fair interobserver reproducibility (ĸ = 0.427). Despite excellent tissue contrast in cCT, the more complex morphological assessments of LAA are challenging and would benefit from supporting quantitative tools or consensus readings between several specialists.

Because stroke recurrence is most probable during the first hours and days after the index stroke, it is important to determine the stroke etiology as soon as possible to prevent stroke recurrence. Compared to MRI, cCT is a faster and more widely available imaging modality with lower cost [15]–[16]. The radiological examination of an acute stroke patient usually includes not only CT of the brain, but also carotid artery CT angiography combined with imaging of the aortic arch [2]. Current prospective ECG-gated CT scans allow extremely low radiation doses [16]. Thus, by extending the scanned area from the carotid arteries and aortic arch down to the level of the LAA would not significantly increase the radiation dose, but may provide a valid method for evaluating the LAA in acute stroke patients [38]. While the main indication for the cCT in these patients would be the detection or exclusion of LAA or other intracardiac thrombi, volumetric and morphological LAA assessments could provide additional information on risk stratifications of stroke recurrence [6].

Our study has several limitations. First, our imaging machinery was relatively old and represented different generations due to the long duration of patient collection. Therefore, the analysed data does not represent the cCT quality of the most modern medical care units. However, the reproducibility of volume measurements was excellent and we can assume that more modern scanners would yield even better results. Second, neither the morphological assessments nor volumetric measurements were performed by experienced cardioradiologists, but by radiology residents or research-oriented Bachelors of medicine who were guided by experts. Therefore, our study shows that LAA and LA volume measurements are robust and reproducible even in inexperienced hands, but assessments of morphological type were more challenging and provided only fair reproducibility between clinically inexperienced observers. On the other hand, the morphological assessment of LAA is not clinically routine, and we assume that the use of more experienced readers would not have changed the results significantly. Third, intra-observer reproducibility was not analysed for LAA morphology assessments. Fourth, reproducibility analyses should be based on data both from symptomatic patients with a wide range of LAA volumes and morphological features, similar to the daily evaluation procedure in clinical practice, and a healthy normal population where the differences in assessed variable are narrower. While our study increased the radiation exposure we saw no justification to use healthy individuals.

In conclusion, LAA and LA volume measurements with cCT are robust and highly reproducible despite differing image quality, variation in LAA or LA volume, and measurements in differing orientations. The visual assessment of LAA morphology had only fair agreement between the readers and would benefit from the development of supporting quantitative tools or consensus readings between several specialists.
